# Microbial Succession and Interactions During the Manufacture of Fu Brick Tea

**DOI:** 10.3389/fmicb.2022.892437

**Published:** 2022-06-23

**Authors:** Meichun Xiang, Jun Chu, Wenjiao Cai, Haikun Ma, Weijing Zhu, Xiaoling Zhang, Jinwei Ren, Lizheng Xiao, Dongbo Liu, Xingzhong Liu

**Affiliations:** ^1^State Key Laboratory of Mycology, Institute of Microbiology, Chinese Academy of Sciences, Beijing, China; ^2^University of Chinese Academy of Sciences, Beijing, China; ^3^State Key Laboratory of Medicinal Chemical Biology, Key Laboratory of Molecular Microbiology and Technology of the Ministry of Education, Department of Microbiology, College of Life Sciences, Nankai University, Tianjin, China; ^4^Department of Tea Science, College of Horticulture, Hunan Agricultural University, Changsha, China

**Keywords:** *Aspergillus cristatus*, Fu Brick tea, pile fermentation, golden flower fungus, microbiota

## Abstract

Fu Brick tea is a very popular post-fermented tea that is known for its “golden flower fungus,” *Aspergillus cristatus*, which becomes the dominant microbe during the maturation process. This study used both culture-dependent methods and high-throughput sequencing to track microbial succession and interactions during the development of the golden flower fungus, a crucial component of the manufacturing process of Fu Brick tea. Among the bacterial communities, *Klebsiella* and *Lactobacillus* were consistently cultured from both fresh tea leaves and in post-fermentation Fu Brick tea. *Methylobacterium, Pelomonas*, and *Sphingomonas* were dominant genera in fresh tea leaves but declined once fermentation started, while *Bacillus, Kluyvera*, and *Paenibacillus* became dominant after piling fermentation. The abundance of *A. cristatus* increased during the manufacturing process, accounting for over 98% of all fungi present after the golden flower bloom in the Fu Brick tea product. Despite their consistent presence during culture work, network analysis showed *Lactobacillus* and *Klebsiella* to be negatively correlated with *A. cristatus*. *Bacillus* spp., as expected from culture work, positively correlated with the presence of golden flower fungus. This study provides complete insights about the succession of microbial communities and highlights the importance of co-occurrence microbes with *A. cristatus* during the manufacturing process of Fu Brick tea.

## Introduction

Fu Brick tea is made from the leaves of *Camellia sinensis* var. *sinensis* after undergoing indigenous microbial fermentation. It is traditionally produced in the Hunan Province of China, and it dates back to the Ming Dynasty (Zhang et al., 2013). Fu Brick tea is consumed widely, especially as a necessary component of milk tea or buttered tea for the nomadic groups in the border regions of southern and western China (Chen, 2000). The health benefits of drinking Fu Brick tea include suppression of fatty acid synthase expression (Chiang et al., 2006), inhibition of lipid, and non-lipid oxidative damage (Jie et al., 2006), as well as anti-obesity (Li et al., 2013; Chen et al., 2018), anti-hyperlipidemia (Fu et al., 2011), and anti-hyperglycemia (Wu et al., 2010). Unlike the other types of fermentation that depend on the enzyme oxidation induced by tea leaves, the post-fermentation process of Fu Brick tea depends on the auto-oxidation and non-enzyme auto-oxidation induced by the indigenous microorganisms on tea leaves (Zheng et al., 2015). The unique hallmark for the quality of Fu Brick tea is the “golden flower fungus,” which earned this name because of the golden cleistothecia produced by the fungus *Aspergillus cristatus* (sexual morph is *Eurotium*-like) (Xu et al., 2011; Chen G. et al., 2017). The formation of small golden spheres throughout the interior of Fu Brick tea during fermentation is crucial for achieving high-quality aroma and taste (Liu et al., 2019).

The manufacture of Fu Brick tea generally involves two separate processes. The first process converts fresh tea leaves to raw dark tea through withering, steaming, rolling, pile fermentation, and drying. The second process (likely occurring in a separate facility) subjects the raw dark tea to blending, steaming, short-term piling, compression into bricks, and fungal fermentation (e.g., golden flower blooming) (Zheng et al., 2015). Pile fermentation and golden flower blooming are two key steps involved in the microbial fermentation process (Liu et al., 2019). Pile fermentation is normally conducted under high humidity (over 86% RH) with temperature above 25°C for up to 40 h to enhance the microbial activities in tea leaves (Zheng et al., 2015). Golden flower blooming occurs with relative humidity under 70% and temperature above 25°C for around 21 days (Wen and Liu, 1991). Several investigations have investigated the dynamics of microbial communities during the Fu Brick manufacturing process that focused solely on either fungi (Li et al., 2017), bacteria (Li et al., 2019), or the pile fermentation process (Li et al., 2018). Microbes other than *A. cristatus* have shown prevalence during the pile fermentation stage, such as fungal species of *Cyberlindnera* and *Candida*, or bacterial species from the *Methylobacterium, Klebsiella*, and *Lactobacillus* genera (Li et al., 2018). This indicates that lactic acid bacteria and yeasts are involved in the fermentation of Fu Brick tea (Zhang et al., 2013). Thus, it is necessary to know the relationships between these prevalent microbes during the manufacturing processes for Fu Brick tea.

In this study, tea samples were collected from different factories and at six stages of processing. By combining culture-dependent methods with high-throughput sequencing, we assessed the dynamics and succession of bacterial and fungal communities in Fu Bricks during the manufacturing process. Specifically, the objectives were to (1) assess the dominant microbes present and (2) investigate microbial interactions at each step of the Fu Brick manufacturing process. Our findings could advance the understanding about microbial fermentation of Fu Brick tea and may offer means for improving the manufacturing process.

## Materials and Methods

### Sample Collection and Preparation

**Figure 1:**
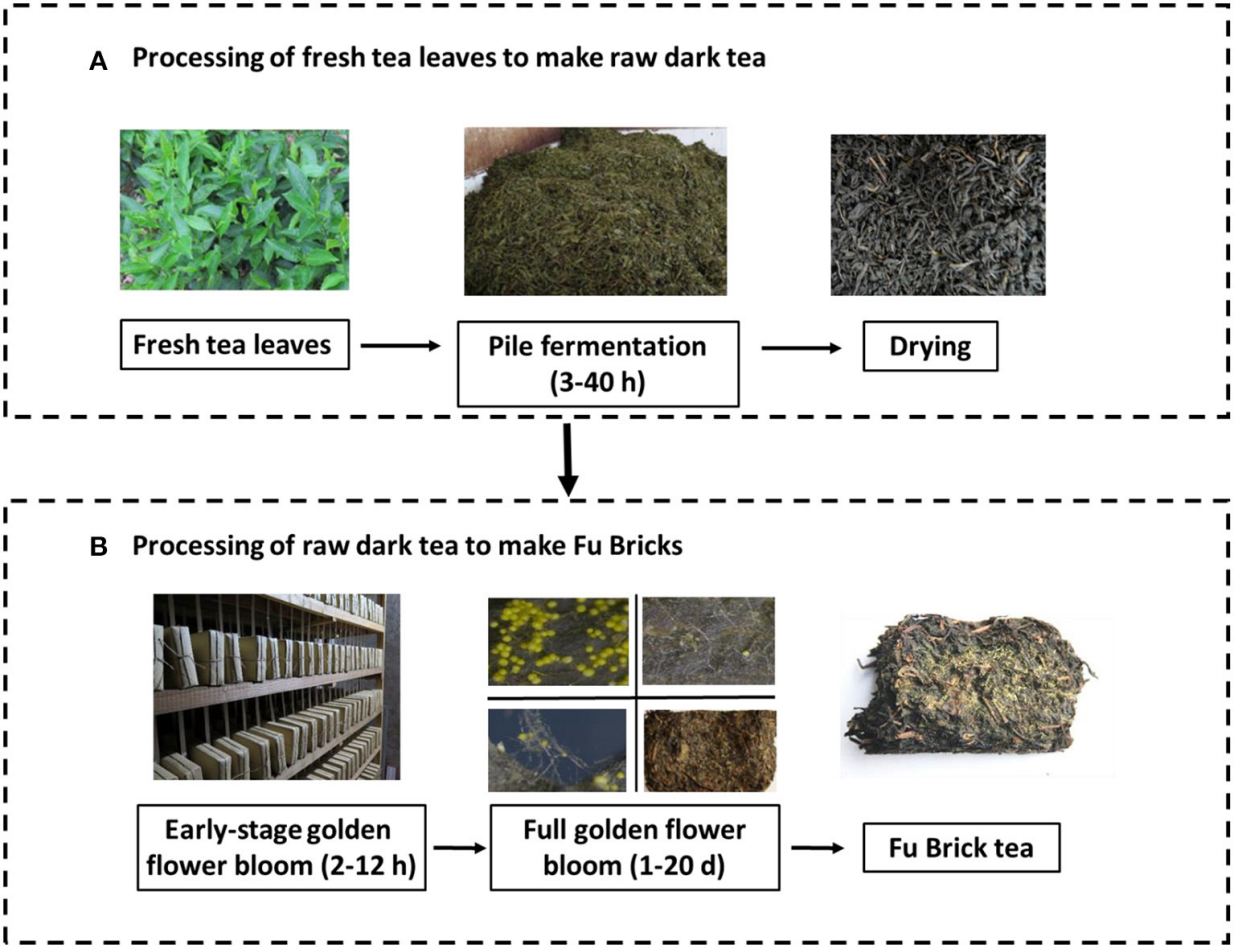
The manufacturing stages of Fu Brick tea. **(A)** The processing of fresh tea leaves to make raw dark tea. **(B)** The processing of raw dark tea to make Fu Bricks.

A total of 90 tea samples were collected at different processing stages from four companies in Anhua, Hunan Province, and one processing facility in Meitan, Guizhou Province, China. The sampled fermentation stages were (in chronological order) as follows: fresh tea (9 samples), pile processing (14 samples), drying (14 samples), early stage golden flower bloom (2–12 h) (22 samples), full golden flower bloom (1–20 days) (23 samples), and post-fermentation (8 samples; Figure 1). The first three stages cover the processing of fresh tea leaves to raw dark tea, and the last three stages cover the processing of raw dark tea to Fu Bricks (Zheng et al., 2015). The pile fermentation and golden flower stages were each sampled at different time points, within each stage, to more accurately assess changes to the microbial community, which were averaged to obtain an overall effect during each stage. The samples were placed in sterile polyethylene bags and transported on ice to the laboratory. All the samples were portioned immediately, with one stored portion at 4°C for culturing of microorganisms and the other stored at −80°C for DNA extraction.

### Culture-Independent Method: DNA Extraction and Sequencing

For each sample, 2.5 g of DNA was extracted with the MoBio PowerSoil^®^ DNA Isolation Kit (MO BIO Laboratories, Inc.) by following the manufacturer's protocols. The extracted DNA was stored at −20°C for further PCR amplification and barcoded amplicon sequencing. Primer sets ITS1-F (5'-CTTGGTCATTTAGAGGAAGTAA-3') and ITS2-R (5'-GCTGCGTTC TTCATCGATGC-3') were used for the amplification of fungal DNA, and primer sets V4-F (5'-GTGCCAGCMGCCGCGGTAA-3') and V4-R (5'-GGACTACHVGGGTWTCTAAT-3') were used for the amplification of bacterial DNA. Both the forward and reverse primers had 6-bp barcodes unique to each sample, which were used to permit multiplexing of samples. The DNA concentrations were adjusted to 20 ng/μl for each sample in the PCR. Each 50 μl PCR solution consisted of 25 μl 2x Taq PCR MasterMix (TIANGEN Company, Beijing), 1.5 μl sense primer (10 μM), 1.5 μl anti-sense primer (10 μM), 3 μl DNA template, and 19 μl ddH_2_O. PCR amplifications were performed in triplicate reactions on a Veriti thermal cycler (Applied Biosystems). PCR amplifications were performed under the following conditions: initial denaturing at 94°C for 4 min, followed by 35 cycles of 94°C for 40 s, 54°C for 45 s (for ITS), or 52°C for 45 s (for V4), 72°C for 50 s, and a final extension step at 72°C for 10 min. Negative controls with no template DNA were also included in PCR amplifications. The products of the triplicate PCRs of each soil sample were pooled and subjected to gel electrophoresis, from which purified amplicons were harvested using the EasyPure Quick Gel Extraction Kit (TransGen, Beijing). The purified amplicons and products encoding different barcodes were combined into a single library for automated cluster generation and paired-end sequencing on the MiSeq system.

Primary data analysis (image analysis, base calling) was performed on a MiSeq instrument. The sequences were analyzed using the Quantitative Insights Into Microbial Ecology (QIIME) toolkit with default parameters for each step (Caporaso et al., 2010). Fungal and bacterial sequences with lengths shorter than 220 nucleotides were removed. *De novo* and reference-based chimera checking were performed, and sequences that were characterized as chimeric were also removed. Bacterial reads were binned into OTUs at the ≥97% sequence similarity level using the open-reference OTU picking protocol in UPARSE-pipeline (Edgar, 2013), and the most abundant sequences from each OTU were taken as representative sequences for the respective OTU. Taxonomic configuration of bacterial OTUs was performed by searching the Basic Local Alignment Search Tool (BLAST) for each representative sequence against a subset of the Silva database (Quast et al., 2013). The fungal sequences were subjected to ≥97% sequence similarity single-linkage clustering using GenBank (Benson et al., 2005) and UNITE (Abarenkov et al., 2012) as reference taxonomic databases.

### Culture-Dependent Method

For each sample, 10 g was homogenized with sterile distilled water using a blender at 12,000 rpm using three 30 s pulses. The homogenate was serially diluted in sterile distilled water and spread onto each of four types of media: MEA (2% malt extract, 0.1% peptone, 2% glucose, 1.8% agar; containing 100 mg/L chloramphenicol and 100 mg/L streptomycin), LA medium (BD Co.), YDP (1% yeast extract, 1% peptone, 2% glucose, 1.8% agar; containing 100 mg/L chloramphenicol), and Gauze No.1 (AOBOX BIOTECHNOLOGY; 25 mg/L nalidixic acid and 50 mg/L cycloheximide) (Liu et al., 1996; Rutiaga-Quiñones et al., 2012). Plates were prepared for each sample and medium in triplicate. The plates were incubated at 25°C for 2 days, and colonies were transferred to new plates to purify and preserve.

For the identification of microbes from pure cultures, morphological examinations were conducted with microscopy. These identifications were then followed by the implementation of DNA-based methods. The genomic DNA was extracted with a simple and rapid “thermolysis” method (Zhang et al., 2010). Briefly, a sterile toothpick was used to transfer mycelia or yeast cells from the colony into 100 μl pure water. The mixture was vortexed thoroughly and centrifuged at 10,000 g for 1 min. The supernatant was removed, and then, 100 μl of lysis solution [EasySelectTM Pichia Expression Kit (Invitrogen, USA)] was added into the microcentrifuge tube. The mixture was then incubated at 85°C for 20–30 min. The extract contained genomic DNA and was stored at −20°C for future use. Multiple primers are used for confirmation of our morphological identifications; they are listed in Supplementary Table 1, and they included the ITS region of fungi (White et al., 1990), the D1/D2 region of yeasts (Boekhout et al., 1995), the 16S rRNA gene of bacteria with degenerate bases (DeLong et al., 2006), and the 16S rRNA gene of actinomycetes without degenerate bases (Lane, 1991). Special primers were used for better differentiating yeast and actinomycetes species based on literature. Each 25 μl PCR solution consisted of 12.5 μl 2x Taq PCR MasterMix (TIANGEN Company, Beijing), 1 μl sense primer (10 μM), 1 μl anti-sense primer (10 μM), 2 μl DNA template, and 8.5 μl ddH_2_O. PCR amplifications were performed on a Veriti Thermal Cycler (Applied Biosystems) under the following conditions: initial denaturing at 94°C for 3 min, followed by 35 cycles at 94°C for 30 s, 55°C for 30 s, and 72°C for 1 min, with a final extension step at 72°C for 10 min (fungi); initial denaturing at 94°C for 3 min, followed by 35 cycles at 94°C for 40 s, 55°C for 30 s, and 72°C for 80 s, with a final extension step at 72°C for 10 min (bacteria); initial denaturing at 94°C for 3 min, followed by 35 cycles at 94°C for 30 s, 55°C for 30 s and 72°C for 1 min, with a final extension step at 72°C for 10 min (yeast); initial denaturing at 94°C for 3 min, followed by 35 cycles at 94°C for 30 s, 55°C for 30 s, and 72°C for 90 s, with a final extension step at 72°C for 10 min (actinomycetes). The PCR products were sent to Omega Genetics Co. Ltd., China, for downstream sequencing, and the obtained sequences were uploaded to GenBank database to search for homologous sequences by BLAST.

### Statistical Analysis

Sequencing data were normalized using total sum scaling (Weiss et al., 2017). Culture-dependent data were shown as counts (CFU/g). All data were checked for homogeneity of variance, and normality was confirmed by inspection of residuals. The overall effects of manufacturing stages on the composition of bacterial and fungal communities were examined using permutational multivariate ANOVA (PERMANOVA) based on a Bray-Curtis dissimilarity matrix (Oksanen et al., 2006). Non-metric multidimensional scaling (NMDS) plots, also based on Bray-Curtis, were used to visualize the separations between manufacturing stages. Linear mixed models were used to examine the effects of the manufacturing process on the Simpson diversities of bacteria and fungi, and the relative abundances of bacterial phyla, fungal classes, *A. cristatus*, yeast, and lactic acid bacteria. In the model, processing stages were used as the fixed factors, and different sampling facilities were used as random factors. Then, *post-hoc* Tukey's tests were used for the pairwise comparisons between all processing stages. The linear mixed model and *post-hoc* Tukey's test described earlier were also used to examine the effects of processing stages on the relative abundances of core microbes (those that dominated in 80% of all samples or had relative abundances >1%). Network analysis based on Pearson's correlations (*P* < 0.05) was used to explore only the relationships between core microorganisms because too few sample sizes would lead to false-positive correlations. The network was constructed using Gephi-0.9.2. Given the likelihood that not all microbes present in our samples would be culturable during the culture-dependent experiments and would result in missing values in the culture-dependent dataset, statistical analysis may not be possible for the culture-dependent dataset. All analyses were performed in R (version 3.0.1, R Development Core Team, 2013).

## Results

### Culture-Independent Analyses Reveal Significant Fluctuations in Microbial Composition

**Figure 2:**
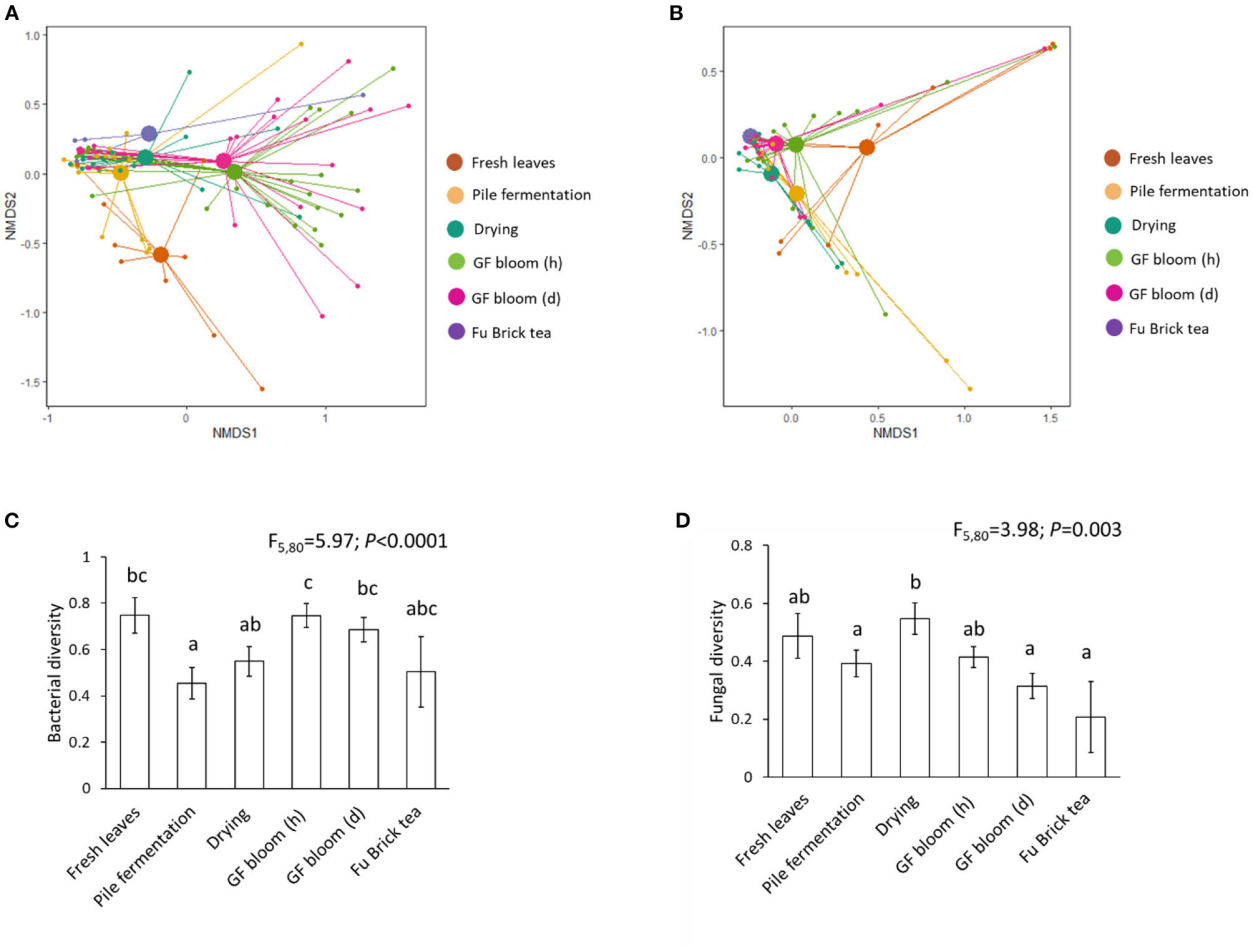
Assessing microbial diversity at different stages of the Fu Brick manufacturing process. Non-metric Multidimensional Scaling (NMDS) plots with Bray-Curtis dissimilarity distances for bacterial **(A)** and fungal **(B)** communities, and bar plots showing statistical results following linear mixed models for bacterial **(C)** and fungal **(D)** diversity. Standard errors from the mean for each stage are shown with each bar, and letters above bars indicate significant differences between manufacturing stages. “GF bloom (h)” is the early stage golden flower bloom and “GF bloom (d)” is the full golden flower bloom.

Fermentation stage one explained 13% of the variances in the bacterial community and 17% in the fungal community (Table 1). Fermentation stage two did not significantly influence the microbial communities. The overall effects of fermentation stages explained 16% of the variances in the bacterial community and 15% in the fungal community (Table 1 and Figure 2). Fresh tea leaves contained more distinct fungal and bacterial communities compared with those present during pile fermentation (Figures 2A,B). Manufacturing stages significantly influenced the Simpson diversity of bacterial and fungal communities (Figures 2C,D). With regard to bacterial diversity, pile fermentation had the lowest level, while early stage golden flower bloom had the highest (Figure 2C). For fungi, the drying stage had the highest amount of diversity, while the Fu Brick tea product (i.e., the final stage of processing) had the lowest (Figure 2D).

**Table 1:** The effects of fermentation stages on the composition of bacterial and fungal communities.

		**Bacterial community**		**Fungal community**	
	**df**	***F***	***R*** ^**2**^	***F***	***R*** ^**2**^
Fermentation stage one	2.36	2.74^*^	0.13	3.69^**^	0.17
Fermentation stage two	2.48	1.05	0.04	0.82	0.03
Fermentation stages	5.84	3.13^***^	0.16	2.92^**^	0.15

*Presented are degrees of freedom, F-values, and R^2^-values from PERMANOVA tests*.

“*,” “**,” and “***” *indicate P < 0.05, 0.01, and 0.001, respectively*.

*“Fermentation stage one” in Figure 1A, and “Fermentation stage two” in Figure 1A. “Fermentation stages” is the overall effects of all fermentation stages*.

**Figure 3:**
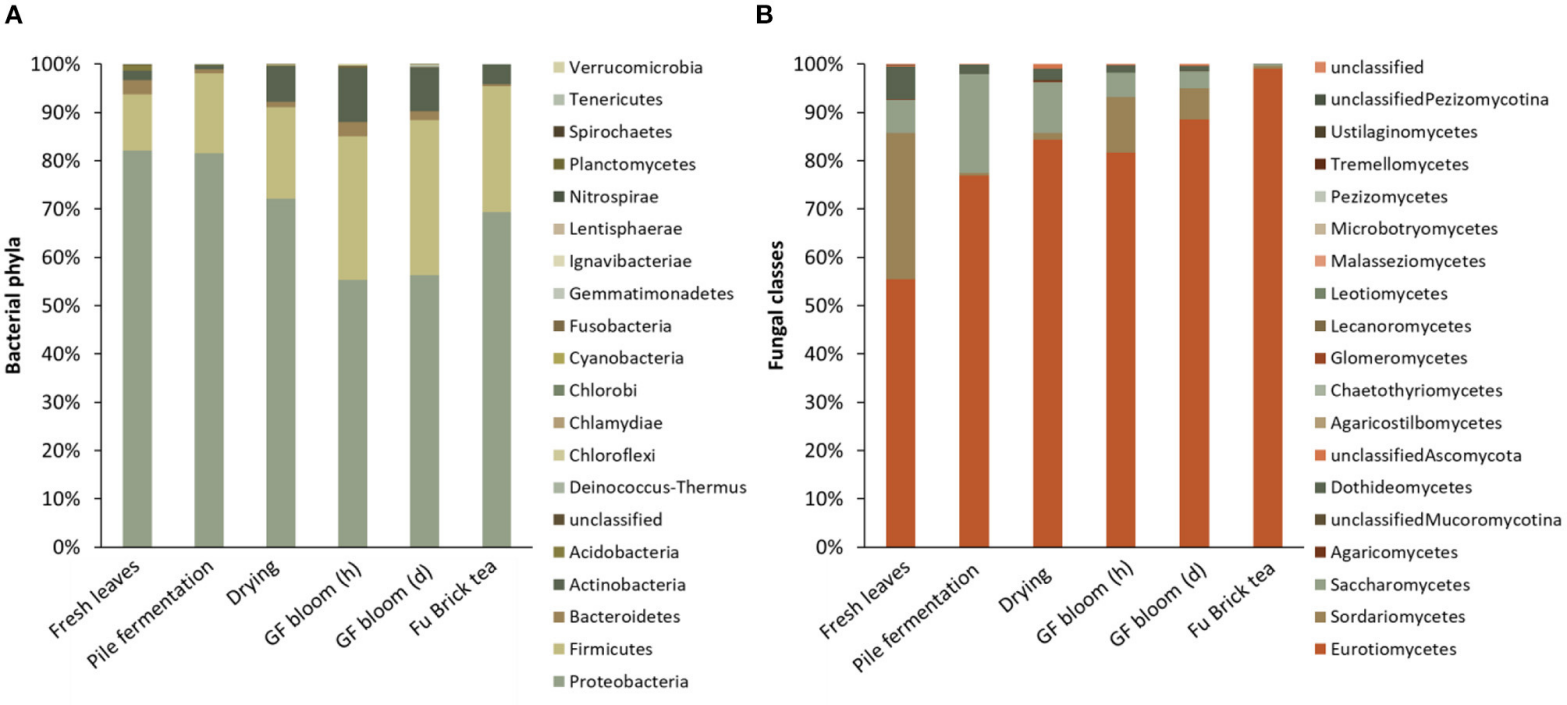
The relative abundances of bacterial phyla **(A)** and fungal classes **(B)** in each examined stage of the Fu Brick manufacturing process. “GF bloom (h)” is the early stage golden flower bloom, and “GF bloom (d)” is the full golden flower bloom.

Across all stages of the manufacturing process, the dominant phyla comprising bacterial communities included the Proteobacteria, Firmicutes, Bacteroidetes, Actinobacteria, and Acidobacteria (Figure 3A and Supplementary Table 2). Fresh tea leaves had the highest relative abundances of Proteobacteria and Acidobacteria and the lowest relative abundance of Firmicutes (Supplementary Table 2). Throughout both fermentation stages, however, the relative abundances of Proteobacteria and Acidobacteria decreased, while the relative abundance of Firmicutes increased (Supplementary Table 2). The final Fu Brick product contained the highest relative abundance of Firmicutes and the lowest relative abundances of Proteobacteria and Acidobacteria (Supplementary Table 2).

At the genus level, the bacterial communities on fresh tea leaves were dominated by *Klebsiella*, followed by *Methylobacterium*. The pile fermentation and drying stages increased the relative abundances of *Klebsiella, Sphingomonas, Paenibacillus*, and *Streptomyces*, which then decreased at the golden flower bloom stages (Table 2). Throughout the fermentation stages, the relative abundance of *Methylobacterium* decreased significantly and reached its lowest level in the Fu Brick product (Table 2). Conversely, the abundances of *Bacillus* and *Pelomonas* species were lowest in fresh tea leaves, but they significantly increased during pile fermentation and reached their highest levels in the Fu Brick product (Table 2).

**Table 2:** The relative abundances of core microbes at each stage of the Fu Brick manufacturing process.

**Core Microbes**	**Df**	**Overall**	**Fresh tea leaves**	**Pile fermentation**	**Drying**	**GF bloom (hrs)**	**GF bloom (days)**	**Fu Brick tea**
*Acinetobacter*	5.80	0.88	0.0034 ± 0.00	0.0024 ± 0.00	0.0081 ± 0.00	0.0331 ± 0.01	0.0364 ± 0.02	0.002 ± 0.00
*Bacillus*	5.80	4.05^**^	0.0392 ± 0.02b	0.082 ± 0.05b	0.0846 ± 0.03ab	0.1711 ± 0.04ac	0.2012 ± 0.04c	0.1304 ± 0.13abc
*Klebsiella*	5.80	7.25^***^	0.3875 ± 0.08a	0.6504 ± 0.06b	0.6028 ± 0.07bc	0.3325 ± 0.06a	0.3816 ± 0.07a	0.581 ± 0.19abc
*Kluyvera*	5.80	1.65	0.0212+0.01	0.0405+0.01	0.0385+0.01	0.067+0.02	0.0661+0.02	0.0905+0.06
*Lactobacillus*	5.80	1.27	0.0621 ± 0.02	0.0747 ± 0.02	0.0711 ± 0.02	0.0288 ± 0.01	0.0396 ± 0.02	0.0768 ± 0.06
*Methylobacterium*	5.80	12.49^***^	0.1073 ± 0.03a	0.0293 ± 0.01b	0.0024 ± 0.00b	0.0041 ± 0.00b	0.0016 ± 0.00b	0.00 ± 0.00b
*Paenibacillus*	5.80	11.90^***^	0.0031+0.00b	0.0025+0.00b	0.0137+0.01bc	0.0534+0.01a	0.0391+0.01a	0.0329+0.03ac
*Pelomonas*	5.80	2.98^*^	0.0459+0.03a	0.0056+0.00b	0.0084+0.00ab	0.0274+0.01ab	0.0125+0.00ab	0.0011+0.00ab
*Pseudomonas*	5.80	0.98	0.0169 ± 0.01	0.0095 ± 0.00	0.0145 ± 0.00	0.0152 ± 0.00	0.0131 ± 0.00	0.0065 ± 0.00
*Sphingomonas*	5.80	2.76^*^	0.0433 ± 0.01a	0.0171 ± 0.01ab	0.0061 ± 0.00b	0.0259 ± 0.01ab	0.0146 ± 0.01ab	0.0004 ± 0.00ab
*Streptomyces*	5.80	4.76^**^	0.0039+0.00bc	0.001+0.00b	0.0146+0.01ac	0.0151+0.00a	0.0171+0.01a	0.0145+0.01ac
*Staphylococcus*	5.80	0.55	0.0031 ± 0.00	0.0023 ± 0.00	0.0035 ± 0.00	0.0092 ± 0.00	0.0146 ± 0.01	0.0051 ± 0.01
*Aspergillus*	5.80	2.48^*^	0.5521 ± 0.10a	0.767 ± 0.08ab	0.8354 ± 0.04ab	0.8122 ± 0.05ab	0.8822 ± 0.04b	0.9901 ± 0.00b
*Cladosporium*	5.80	3.68^**^	0.0156 ± 0.01a	0.0059 ± 0.00ab	0.0004 ± 0.00b	0.0002 ± 0.00b	0.0002 ± 0.00b	0.0001 ± 0.00ab
*Cyberlindnera*	5.80	1.34	0.0696 ± 0.04	0.202 ± 0.08	0.1041 ± 0.04	0.0498 ± 0.02	0.036 ± 0.01	0.0013 ± 0.00
*Fusarium*	5.80	0.97	0.0016 ± 0.00	0.0011 ± 0.00	0.0026 ± 0.00	0.0012 ± 0.00	0.0012 ± 0.00	0.0006 ± 0.00
*Ilyonectria*	5.80	0.76	0.0015 ± 0.00	0.0012 ± 0.00	0.002 ± 0.00	0.0012 ± 0.00	0.0012 ± 0.00	0.0003 ± 0.00
*Metacordyceps*	5.80	0.32	0.0011 ± 0.00	0.0006 ± 0.00	0.0005 ± 0.00	0.0007 ± 0.00	0.0008 ± 0.00	0.0001 ± 0.00
*Penicillium*	5.80	2.43^*^	0.0026 ± 0.00ab	0.001 ± 0.00a	0.007 ± 0.00b	0.0045 ± 0.00ab	0.0023 ± 0.00ab	0.0005 ± 0.00ab
*Purpureocillium*	5.80	3.61^**^	0.2863+0.13a	0.0008+0.00ab	0.0001+0.00b	0.1079+0.04ab	0.0573+0.04b	0.0037+0.00ab
*Uwebraunia*	5.80	1.62	0.0295+0.02	0.0062+0.00	0.0125+0.01	0.0094+0.00	0.0062+0.00	0.00+0.00

*“Core microbes” are listed at the genus level and found in ≥80% of all samples or had an average relative abundance of >1%. “Df” is the degree of freedom. “Overall” is the effect of fermentation stages following a linear mixed model, presented as F-values*.

*, **, and *** *indicate P < 0.05, 0.01, and 0.001, respectively*.

*“GF bloom (h)” is the early stage golden flower bloom and “GF bloom (d)” is the full golden flower bloom. In each manufacturing stage, values are mean + se of the relative abundances of microbial genera. Letters following mean ± se indicate significant differences in the mean (P < 0.05)*.

The dominant classes within fungal communities throughout the manufacturing process consisted of Eurotiomycetes, Sordariomycetes, Saccharomycetes, and Dothideomycetes (Figure 3B and Supplementary Table 2). Fresh tea leaves contained the lowest relative abundance of Eurotiomycetes and the highest relative abundances of Dothideomycetes and Sordariomycetes (Supplementary Table 3). As subsequent processing stages progressed, the relative abundance of Eurotiomycetes increased and the relative abundances of Dothideomycetes and Sordariomycetes decreased, with Fu Bricks containing the highest relative abundance of Eurotiomycetes and the lowest relative abundances of Dothideomycetes and Sordariomycetes (Supplementary Table 3). The relative abundance of Saccharomycetes first increased, and became highest in the pile fermentation stage, and then decreased, with Fu Bricks containing the lowest relative abundance of Saccharomycetes (Supplementary Table 3).

So far, *Aspergillus*, especially *A. cristatus*, was the dominant fungal genus, which increased significantly across all processing stages from fresh tea leaves (low levels) to comprise 98% of the Fu Brick product (Table 2). Other fungal genera predominated in fresh tea leaves, such as *Penicillium, Purpureocillium*, and *Cladosporium*. As fermentation progressed, their relative abundances fluctuated. By the drying stage, *Penicillium* and *Purpureocillium* species had the lowest relative abundances (Table 2). Both fermentation stages caused consistent decreases in the relative abundance of *Cladosporium* fungi with the Fu Brick product containing the lowest relative abundance of this genus (Table 2). Despite being considered a dominant fungal class, no genus within the Saccharomycetes satisfied the two standards of core microbes for this study.

### Culture-Dependent Analyses Support the Fluctuation of Dominant Microbes During the Manufacturing Process

**Figure 4:**
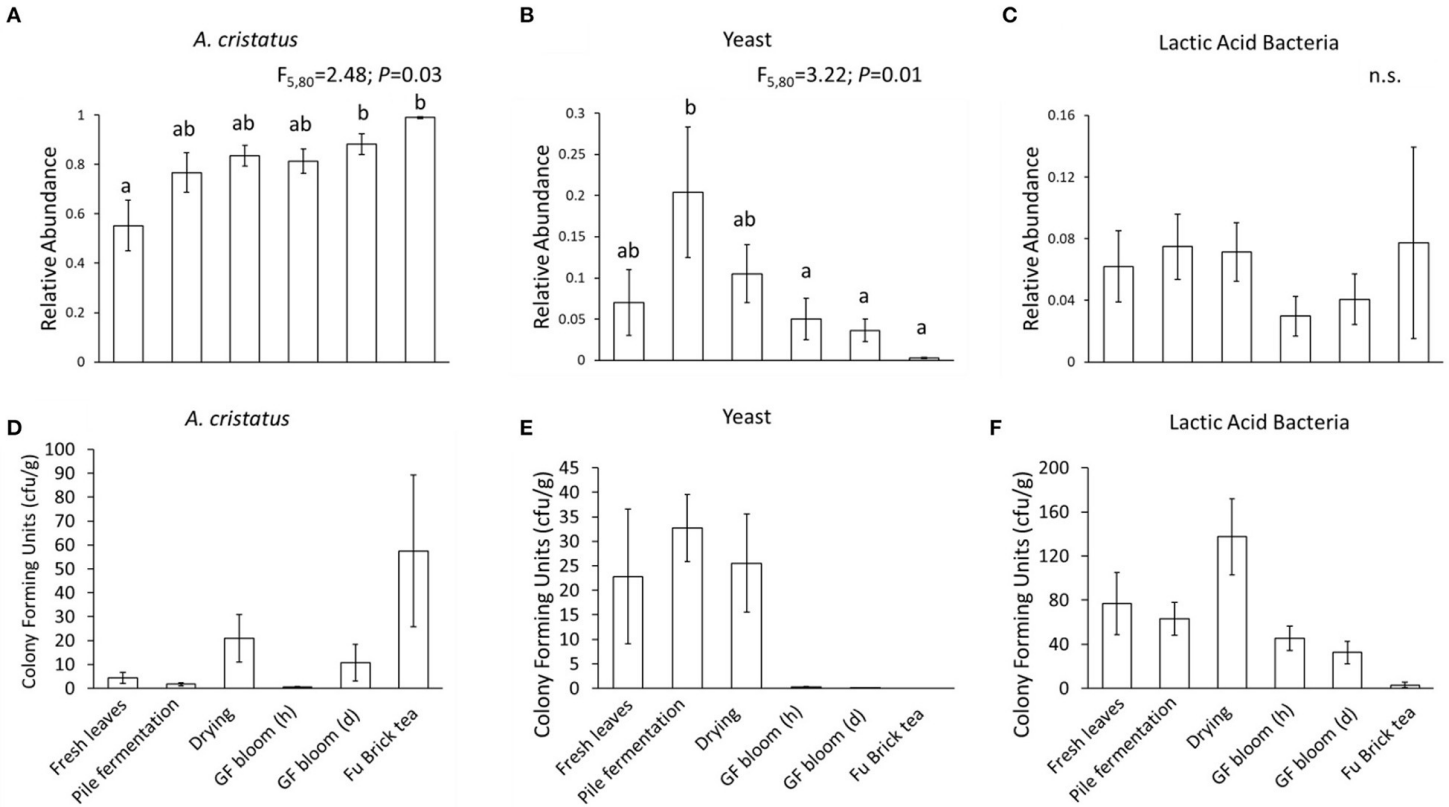
Bar plots showing the relative abundances **(A–C)** and colony forming units **(D–F)** of *A. cristatus* golden flower fungus, yeasts, and lactic acid bacteria, respectively, based on culture-dependent analysis at each examined stage of Fu Brick manufacturing. Standard errors from the mean for each stage are shown with each bar. Statistical results following linear mixed models (A-C) are presented above each panel. “n.s.” indicates no significant effect. Letters above bars indicate significant differences between manufacturing stages. “GF bloom (h)” is the early stage golden flower bloom and “GF bloom (d)” is the full golden flower bloom.

In this study, we classified the microbes as *A. cristatus*, yeasts, or lactic acid bacteria based on their colony forming units (cfu) (Figure 4). Overall, we found that the culture-based data at each processing stage supported the sequencing data: fresh tea leaves contained the lowest abundance of *A. cristatus* cfu, which increased along the manufacturing process to attain the greatest abundance in the Fu Brick product (Figures 4A,E). Yeasts were abundant in fresh tea leaves, but during fermentation, their numbers decreased. This fate continued through the final Fu Brick stage where they had the lowest abundance (Figures 4B,F). The culture-dependent data showed that all isolated bacteria cfu (including the lactic acid bacteria) were highest at the drying stage and lowest in the Fu Brick product (Figure 4G), while the sequencing data suggested that the relative abundances of lactic acid bacteria did not significantly fluctuate during the manufacturing process (Figure 4C).

### Network Analyses Reveal Important Relationships Among Core Microbial Groups

**Figure 5:**
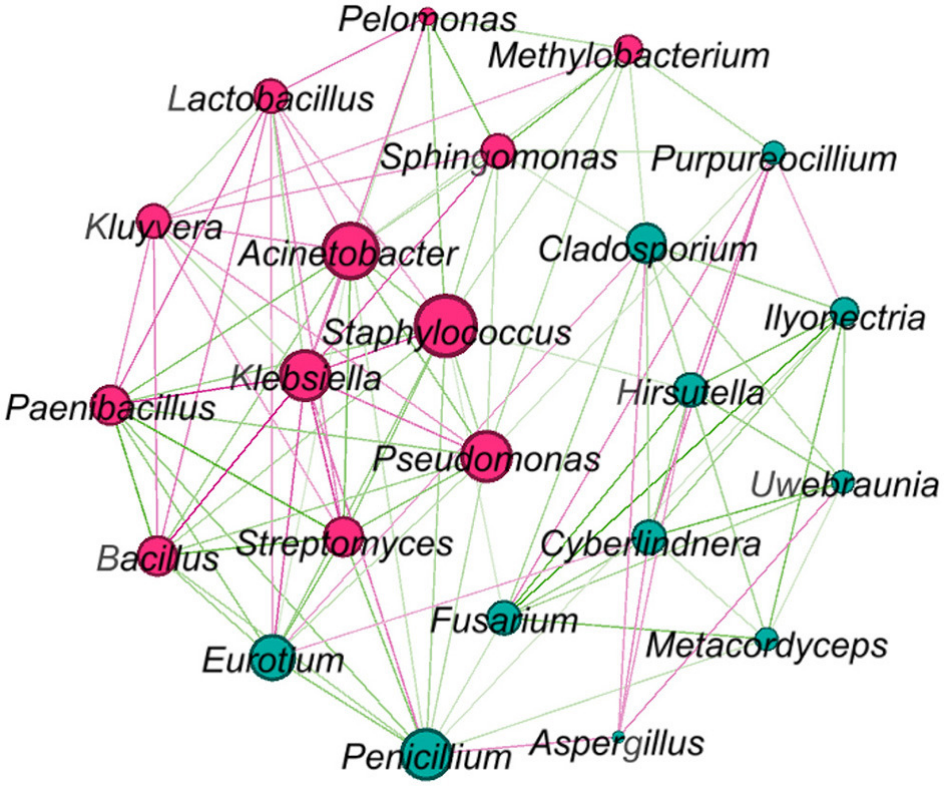
Network graph showing relationships between core microbes (bacteria and fungi) presented in Table 2. Larger nodes indicate higher connections with other genera. Red connections indicate negative correlations, while green connections indicate positive correlations. Pink nodes indicate bacterial genera and green nodes indicate fungal genera.

Network analysis was conducted for the microbes that presented over 80% of all samples (Figure 5). *Klebsiella, Staphylococcus*, and *Acinetobacter* were in the core of the network, *Klebsiella* negatively correlated with most other microbes, while *Staphylococcus* and *Acinetobacter* had both positive and negative relationships with other microbes. *Bacillus, Penicillium*, and *Streptomyces* positively correlated with *A. cristatus*, but *Lactobacillus, Klebsiella*, and *Cyberlindnera* negatively correlated with this fungus.

## Discussion

Many beneficial qualities of Fu Brick tea exist because of fermentation microbes and their metabolites (Zheng et al., 2015), especially the golden flower fungus, which is inherently present on fresh tea leaves and becomes the dominant organism solely through indigenous microbial succession during the manufacturing process. In this study, both the high-throughput sequencing and culture-dependent data revealed drastic changes to microbial community structure that continued to fluctuate throughout each stage of the manufacturing process. Many bacterial genera that were present in fresh tea leaves were lost during the manufacturing process. However, the relative abundance of *Bacillus* bacteria increased as each stage of manufacturing progressed. In fact, this bacterial genus was observed to be positively correlated with the increase in abundance of *A. cristatus*. The positive correlation between *Bacillus* and *A. cristatus* indicated that these bacteria might facilitate and enhance the development of the golden flower fungus. Although both *Penicillium* and *Aspergillus* were the dominant genera in fresh tea leaves, only the abundance of *A. cristatus* continued to increase at each stage of the manufacturing process until it became the dominant microbe, an indicator of high-quality Fu Brick tea.

The succession in bacterial and fungal communities of Fu Brick tea has been extensively investigated (Xu et al., 2011; Li et al., 2017, 2018, 2019). However, those studies mainly correlated microbiota with the biochemical components of Fu Brick tea. In this study, we focused not only on the golden flower fungus but also on the co-occurrence of fungi and bacteria, thus providing a comprehensive picture of the fluctuation of the microbiome during the manufacturing processes. Moreover, our study explored microbial community changes at different stages of the Fu Brick manufacturing process. In other studies, samples were collected at a single time point, such as in the pile fermentation stage, which lasts for 24 h, or at different time points but only in the golden flower blooming stage, which takes around 20 days (Xu et al., 2011; Zheng et al., 2015; Li et al., 2019). Not including microbial communities from the first fermentation stage (piling), in addition to the second fermentation stage (golden flower bloom), leads to an unbalanced representation of the manufacturing process where the pile fermentation microbes would be overlooked, and the golden flower fermentation microbes would be over-represented (as if they are present at all stages). In this study, we offer a more accurate representation of the dynamic microbial changes that occur over the entire processing of Fu Brick tea by sampling several time points in both stages of fermentation. Due to our thorough sampling, we illustrated the variable succession of microbial communities throughout the Fu Brick manufacturing process. More importantly, we showed that the golden flower blooming stage had the greatest influence on the composition of bacterial communities and that abundance of one bacterial genus, in particular, *Bacillus*, was directly correlated with the increase of *A. cristatus*, the golden flower fungus.

Previous investigations associating the Fu Brick microbial community with biochemical components of the tea provided essential information to support our observations (Li et al., 2017, 2018, 2019). For example, *Klebsiella* had the highest relative abundance on both fresh tea leaves and the final Fu Brick tea product despite decreases in abundance during the other processing stages. Previous studies have shown the predominant role of *Klebsiella* during the Fu Brick manufacturing process (Liu et al., 2019), associating this bacterium with the metabolism of biochemical components in Fu Bricks, such as polyphenol, caffeine, and gallocatechin gallate (Li et al., 2019), as well as the process of saccharification by producing ethanol (Doran et al., 1994; Zhou and Ingram, 2001; Golias et al., 2002) and butanediol (Cao et al., 1997; Sun et al., 2009; Moon et al., 2018), especially in co-culture with fungi (Yu et al., 1985; Cao et al., 1997). Other bacterial genera (*Methylobacterium, Pelomonas*, and *Sphingomonas*) had high relative abundances on fresh tea leaves, which greatly decreased during the Fu Brick manufacturing process. *Methylobacterium* is known for the production of poly-β-hydroxybutyrate (Bourque et al., 1992; Yellore et al., 2000; Ghatnekar et al., 2002) and was identified as one of the core bacteria during the pile fermentation processing stage (Liu et al., 2019). It is also one of the predominant genera of raw Pu'er tea, which is another type of fermented tea derived from the leaves of *C. sinensis* var. *sinensis* (Ma et al., 2017). Although *Pelomonas* was only recently discovered in Fu Brick tea (Li et al., 2018, 2019), it has been found in other fermented foods, such as kefir grains (Gao et al., 2013), Shaoxing liquor (Chen et al., 2020), and fermented grape, where it was associated with the production of lactic and acetic acids (del Carmen Portillo and Mas, 2016). Thus, the presence of *Pelomonas* indicates the flavor of the food products. Three bacterial genera (i.e., *Bacillus, Kluyvera*, and *Paenibacillus*) increased in abundance to become dominant during the manufacturing process of Fu Brick tea. *Bacillus* can produce pectinase (Rehman et al., 2015; Uzuner and Cekmecelioglu, 2015) and oxidase polyphenol (Mohammad and Alireza, 2007), which are important for oxidating the major catechins into theaflavin (Zhu et al., 2015). *Bacillus* has been linked to the flavor profile of Fu Brick (Zheng et al., 2015; Liu et al., 2019). Moreover, a recent study has found that *Bacillus* correlated with 44 metabolites of Fu Brick tea, highlighting the importance of *Bacillus* in manufacturing Fu Brick tea (Xia et al., 2022). Although *Pseudomonas* were not significantly changed between different manufacturing stages in our study, they were revealed by metagenomic methods as the most important bacteria associated with 35 probiotics in Fu Brick tea (Wang et al., 2021). Other dominant bacteria, such as *Paenibacillus*, were reported to be one of the key microbes in the manufacture of other traditionally fermented foods, including Pu'er tea (Oh et al., 2008; Kim et al., 2009; Zhao et al., 2015).

With regard to fungal genera, *A. cristatus* accounted for about 50% of all fungi in fresh tea leaves and increased up to 98% in the Fu Brick tea product. This pattern was further confirmed by the culture-dependent data, whereby *A. cristatus* was the dominant fungus. Some species of *Aspergillus* are commonly used in the traditional fermentation of foods where they reportedly degrade plant cell walls, produce peroxiredoxins, and contribute to the oxidation of catechins, such as during the manufacture of Pu'er tea (Chen A. J. et al., 2017). As the uniquely dominant fungus in Fu Brick tea, *A. cristatus* was reported to be responsible for the production of polyphenol oxidase, cellulase, and pectinase (Li et al., 2018), as well as serving as an important probiotic that reduces obesity in humans (Kang et al., 2019). *Purpureocillium* and *Cyberlindnera* were also dominant fungi in the fresh tea leaves used for Fu Brick tea, accounting for 28 and 6% of all fungal genera observed, respectively. The presence of *Purpureocillium* fungi in Fu Brick tea has not been previously reported; however, they have been observed in other fermented foods, such as beans (Serra et al., 2019), Chinese Paocai (Xiao et al., 2018), and liquor (Song et al., 2017), as well as being associated with the production of amino acids (Xiao et al., 2018). *Cyberlindnera* has been reported as one of the core microbes observed during the piling fermentation process to create raw dark tea (Li et al., 2017; Liu et al., 2019). As a genus of yeast, *Cyberlindnera* may facilitate the bioconversion of sugars into ethanol, CO_2_, and other metabolites during the piling fermentation process of Fu Brick manufacture (Tofalo et al., 2019).

Generalized relationships between bacteria and fungi in Fu Brick tea have been reported (Rui et al., 2019). Our study provided further information about the dominant bacterial genera that are highly correlated with fermentation fungi (i.e., *A. cristatus*) throughout the entire manufacturing process. As the golden flower fungus has been extensively credited for improving the flavor and quality of Fu Brick tea in the fermentation process (Angayarkanni et al., 2002; Wulandari et al., 2011; Yao et al., 2017; Jiang et al., 2018), it is important to identify any microbes that may facilitate its growth and development. However, the mechanisms underscoring these relationships between *A. cristatus* and dominant Fu Brick bacteria remain unknown. Understanding these relationships throughout the entire manufacturing process should help improve tea quality and streamline the Fu Brick manufacturing process.

## Conclusion

The succession of Fu Brick core microbes over time indicated that certain bacterial and yeast genera do not survive the fermentation stages, especially in the presence of the golden flower fungus, *A. cristatus*, that greatly increases its infiltration of the tea product during fermentation. However, some bacterial and yeast genera do survive and increase in direct proportion with *A. cristatus*. Although the golden flower fungus has long been considered the most important microbial component in Fu Brick tea, other microbes (e.g., *Bacillus*) that positively correlated with *A. cristatus* growth also deserve further investigation to explore the causal relationships between fermentation-thriving organisms in Fu Bricks. Associating microbial succession and interactions that affect the biochemical components of Fu Brick tea could improve forecasting of tea quality and improve the manufacturing process.

## Data Availability Statement

The datasets presented in this study can be found in online repositories. The names of the repository/repositories and accession number(s) can be found below: https://osf.io/2cbq4, https://osf.io/2cbq4.

## Author Contributions

MX and XL designed the experiment. MX, JC, and WC took the tea samples. WZ and XZ isolated microbes from the samples. WC and HM analyzed the data. XL and MX lead the writing of the manuscript. All authors revised and give comments to the manuscripts. All authors contributed to the article and approved the submitted version.

## Conflict of Interest

The authors declare that the research was conducted in the absence of any commercial or financial relationships that could be construed as a potential conflict of interest.

## Publisher's Note

All claims expressed in this article are solely those of the authors and do not necessarily represent those of their affiliated organizations, or those of the publisher, the editors and the reviewers. Any product that may be evaluated in this article, or claim that may be made by its manufacturer, is not guaranteed or endorsed by the publisher.
