# Supplementary material for: Microbial Succession and Interactions During the Manufacture of Fu Brick Tea
Source: Front Microbiol. 2022 Jun 23;13:892437. doi: 10.3389/fmicb.2022.892437 (PMC9261264; doi:10.3389/fmicb.2022.892437)
Supplement: Supplementary file 1 [file Table_1.DOCX]

Supplementary Material

**Microbial succession and interactions during the manufacture of Fu Brick tea**

**Table S1** primers used in culture-dependent method

| Name | Sequence | Reference |
| --- | --- | --- |
| ITS4 | 5'-TCCTCCGCTTATTGATATGC-3' | White et al. 1990 |
| ITS5 | 5'-GGAAGTAAAAGTCGTAACAAGG-3' |  |
| NL1 | 5'-GCATATCAATAAGCGGAGGAAAAG-3' | Boekhout et al. 1995 |
| NL4 | 5'-GGTCCGTGTTTCAAGACGG-3' |  |
| 27F | 5'-AGAGTTTGATCMTGGCTCAG-3' | DeLong et al. 2006 |
| 1492R | 5'-TACGGYTACCTTGTTACGACTT-3' |  |
| 27F | 5'-AGAGTTTGATCCTGGCTCAG-3' | Lane 1991 |
| 1492R | 5'-GGTTACCTTGTTACGACTT-3' |  |

**Table S2** The relative abundance of bacterial phyla in each fermentation stage

| Bacteria phyla | Df | Overall | Fresh tea leaves | Pile fermentation | Drying | GF bloom (hrs) | GF bloom (days) | Fu Brick tea |
| --- | --- | --- | --- | --- | --- | --- | --- | --- |
| Acidobacteria | 5,80 | 3.25* | 0.0116+0.01a | 0.0008+0.00b | 0.0009+0.00b | 0.0018+0.00ab | 0.0007+0.00b | 0.00+0.00ab |
| Actinobacteria | 5,80 | 1.86 | 0.0205+0.01 | 0.0089+0.00 | 0.0758+0.03 | 0.1158+0.02 | 0.0925+0.03 | 0.0406+0.04 |
| Bacteroidetes | 5,80 | 1.87 | 0.0288+0.02 | 0.009+0.00 | 0.0113+0.01 | 0.0303+0.01 | 0.019+0.00 | 0.0047+0.00 |
| Chlamydiae | 5,80 | 0.96 | 0.00+0.00 | 0.00+0.00 | 0.00+0.00 | 0.00+0.00 | 0.00+0.00 | 0.00+0.00 |
| Chlorobi | 5,80 | 0.53 | 0.00+0.00 | 0.00+0.00 | 0.00+0.00 | 0.00+0.00 | 0.00+0.00 | 0.00+0.00 |
| Chloroflexi | 5,80 | 1.98 | 0.0001+0.00 | 0.00+0.00 | 0.0011+0.00 | 0.002+0.00 | 0.0018+0.00 | 0.00+0.00 |
| Cyanobacteria | 5,80 | 0.37 | 0.00+0.00 | 0.0001+0.00 | 0.0003+0.00 | 0.0002+0.00 | 0.0005+0.00 | 0.00+0.00 |
| Deinococcus-Thermus | 5,80 | 0.73 | 0.0008+0.00 | 0.0001+0.00 | 0.0004+0.00 | 0.0012+0.00 | 0.004+0.00 | 0.00+0.00 |
| Firmicutes | 5,80 | 5.45** | 0.1176+0.04a | 0.165+0.05ac | 0.1907+0.04ac | 0.3085+0.05b | 0.3314+0.06b | 0.261+0.17c |
| Fusobacteria | 5,80 | 1.65 | 0.00+0.00 | 0.00+0.00 | 0.0001+0.00 | 0.00+0.00 | 0.00+0.00 | 0.00+0.00 |
| Gemmatimonadetes | 5,80 | 0.85 | 0.00+0.00 | 0.00+0.00 | 0.00+0.00 | 0.0001+0.00 | 0.0001+0.00 | 0.00+0.00 |
| Ignavibacteriae | 5,80 | 0.93 | 0.00+0.00 | 0.00+0.00 | 0.00+0.00 | 0.00+0.00 | 0.00+0.00 | 0.00+0.00 |
| Lentisphaerae | 5,80 | 0.53 | 0.00+0.00 | 0.00+0.00 | 0.00+0.00 | 0.00+0.00 | 0.00+0.00 | 0.00+0.00 |
| Nitrospirae | 5,80 | 0.43 | 0.00+0.00 | 0.00+0.00 | 0.00+0.00 | 0.00+0.00 | 0.00+0.00 | 0.00+0.00 |
| Planctomycetes | 5,80 | 1.09 | 0.00+0.00 | 0.00+0.00 | 0.0001+0.00 | 0.0008+0.00 | 0.0003+0.00 | 0.00+0.00 |
| Proteobacteria | 5,80 | 8.51*** | 0.8285+0.04a | 0.8188+0.05a | 0.73+0.06ac | 0.5754+0.06b | 0.5802+0.07b | 0.6933+0.21bc |
| Spirochaetes | 5,80 | 0.91 | 0.00+0.00 | 0.00+0.00 | 0.00+0.00 | 0.00+0.00 | 0.00+0.00 | 0.00+0.00 |
| Tenericutes | 5,80 | 4.69** | 0.00+0.00b | 0.00+0.00 b | 0.00+0.00 b | 0.00+0.00 b | 0.00+0.00 b | 0.0004+0.00a |
| Verrucomicrobia | 5,80 | 3.14* | 0.00+0.00ab | 0.00+0.00b | 0.00+0.00ab | 0.0002+0.00a | 0.00+0.00b | 0.00+0.00ab |
| unclassified | 5,80 | 4.79** | 0.0009+0.00ab | 0.0006+0.00b | 0.0006+0.00b | 0.0025+0.00a | 0.0009+0.00b | 0.00+0.00ab |

“Df” is degree of freedom.“Overall” is the effect of fermentation stages following a linear mixed model, presented as F values. *, **,*** indicate P < 0.05, 0.01, 0.001, respectively. “GF bloom (h)” is early stage golden flower bloom, “GF bloom (d)” is full golden flower bloom. In each manufacturing stage, values are mean + se of the relative abundances of microbial genera. Letters following mean + se indicate significant differences in the mean (P < 0.05).

**Table S3** The relative abundance of fungal class in each fermentation stage

| Fungal class | Df | Overall | Fresh tea leaves | Pile fermentation | Drying | GF bloom (hrs) | GF bloom (days) | Fu Brick tea |
| --- | --- | --- | --- | --- | --- | --- | --- | --- |
| Agaricomycetes | 5,80 | 1.05 | 0.0003+0.00 | 0.0002+0.00 | 0.003+0.00 | 0.0008+0.00 | 0.0002+0.00 | 0.0005+0.00 |
| Agaricostilbomycetes | 5,80 | 1.60 | 0.00+0.00 | 0.00+0.00 | 0.0002+0.00 | 0.0001+0.00 | 0.00+0.00 | 0.00+0.00 |
| Chaetothyriomycetes | 5,80 | 3.43** | 0.0001+0.00a | 0.00+0.00b | 0+0.00b | 0.00+0.00b | 0.00+0.00b | 0.00+0.00ab |
| Dothideomycetes | 5,80 | 3.71** | 0.0682+0.03a | 0.0196+0.01b | 0.0236+0.01ab | 0.0147+0.01b | 0.0104+0.00b | 0.0002+0.00b |
| Eurotiomycetes | 5,80 | 2.50* | 0.5552+0.10a | 0.7685+0.08ab | 0.8439+0.04ab | 0.8173+0.05ab | 0.885+0.04b | 0.9906+0.00b |
| Glomeromycetes | 5,80 | 0.68 | 0.0002+0.00 | 0.0001+0.00 | 0.00+0.00 | 0.0001+0.00 | 0.0001+0.00 | 0.00+0.00 |
| Lecanoromycetes | 5,80 | 1.64 | 0.00+0.00 | 0.00+0.00 | 0.00+0.00 | 0.00+0.00 | 0.00+0.00 | 0.00+0.00 |
| Leotiomycetes | 5,80 | 2.20 | 0.00+0.00 | 0.00+0.00 | 0.00+0.00 | 0.00+0.00 | 0.00+0.00 | 0.00+0.00 |
| Malasseziomycetes | 5,80 | 1.62 | 0.00+0.00 | 0.00+0.00 | 0.00+0.00 | 0.00+0.00 | 0.00+0.00 | 0.00+0.00 |
| Microbotryomycetes | 5,80 | 3.09* | 0.00+0.00a | 0.00+0.00a | 0.00+0.00ab | 0.00+0.00b | 0.00+0.00b | 0.00+0.00ab |
| Pezizomycetes | 5,80 | 1.26 | 0.00+0.00 | 0.00+0.00 | 0.0001+0.00 | 0.00+0.00 | 0.00+0.00 | 0.00+0.00 |
| Saccharomycetes | 5,80 | 1.37 | 0.0699+0.04ab | 0.2036+0.08b | 0.1051+0.04ab | 0.0501+0.02a | 0.0362+0.01a | 0.0027+0.00a |
| Sordariomycetes | 5,80 | 3.86** | 0.3017+0.13a | 0.0069+0.00b | 0.0138+0.00b | 0.1143+0.04ab | 0.0642+0.04ab | 0.0054+0.00ab |
| Tremellomycetes | 5,80 | 1.51 | 0.0001+0.00 | 0.00+0.00 | 0.0001+0.00 | 0.00+0.00 | 0.00+0.00 | 0.00+0.00 |
| Ustilaginomycetes | 5,80 | 1.85 | 0.0021+0.00 | 0.00+0.00 | 0.00+0.00 | 0.00+0.00 | 0.00+0.00 | 0.00+0.00 |
| unclassified Ascomycota | 5,80 | 0.96 | 0.0017+0.00 | 0.0004+0.00 | 0.008+0.01 | 0.0024+0.00 | 0.0034+0.00 | 0.0001+0.00 |
| unclassified Mucoromycotina | 5,80 | 4.14** | 0.0001+0.00b | 0.0003+0.00b | 0.002+0.00a | 0.00+0.00b | 0.0001+0.00b | 0.0003+0.00ab |
| unclassified Pezizomycotina | 5,80 | 0.90 | 0.00+0.00 | 0.00+0.00 | 0.00+0.00 | 0.00+0.00 | 0.00+0.00 | 0.00+0.00 |
| unclassified | 5,80 | 0.64 | 0.0004+0.00 | 0.0003+0.00 | 0.0002+0.00 | 0.0002+0.00 | 0.0002+0.00 | 0.00+0.00 |

“Df” is degree of freedom.“Overall” is the effect of fermentation stages following a linear mixed model, presented as F values. *, **,*** indicate P < 0.05, 0.01, 0.001, respectively. “GF bloom (h)” is early stage golden flower bloom, “GF bloom (d)” is full golden flower bloom. In each manufacturing stage, values are mean + se of the relative abundances of microbial genera. Letters following mean + se indicate significant differences in the mean (P < 0.05).
